# Instrumental and socioemotional communications in doctor-patient interactions in urban and rural clinics

**DOI:** 10.1186/1472-6963-13-261

**Published:** 2013-07-08

**Authors:** Kristen Desjarlais-deKlerk, Jean E Wallace

**Affiliations:** 1 Department of Sociology, University of Calgary, 2500 University Drive NW, Calgary, AB T2N 1N4, Canada

**Keywords:** Physicians, Family practice, Rural clinics, Urban clinics, Patient-physician communication, Patient-physician interaction

## Abstract

**Background:**

Location of practice, such as working in a rural or urban clinic, may influence how physicians communicate with their patients. This exploratory pilot study examines the communication styles used during doctor-patient interactions in urban and rural family practice settings in Western Canada.

**Methods:**

We analyzed observation and interview data from four physicians practicing in these different locations. Using a grounded theory approach, communications were categorized as either instrumental or socioemotional. Instrumental communication refers to “cure-oriented interactions” and tends to be more task-oriented focusing on the patient’s health concerns and reason for the appointment. In contrast, socioemotional communication refers to more “care-oriented interactions” that may make the patient feel comfortable, relieve patient anxiety and build a trusting relationship.

**Results:**

The physicians in small, rural towns appear to know their patients and their families on a more personal level and outside of their office, and engage in more socioemotional communications compared to those practicing in suburban clinics in a large urban centre. Knowing patients outside the clinic seems to change the nature of the doctor-patient interaction, and, in turn, the doctor-patient relationship itself. Interactions between urban doctors and their patients had a mixture of instrumental and socioemotional communications, while interactions between rural doctors and their patients tended to be highly interpersonal, often involving considerable socioemotional communication and relationship-building.

**Conclusions:**

Despite the different ways that doctors and patients communicate with each other in the two settings, rural and urban doctors spend approximately the same amount of time with their patients. Thus, greater use of socioemotional communication by rural doctors, which may ease patient anxiety and increase patient trust, did not appear to add extra time to the patient visit. Research suggests that socioemotional communication may ultimately lead to better patient outcomes, which implies that health differences between rural and urban settings could be linked to differences in doctor-patient communication styles.

## Background

Examining doctor-patient interactions is important for understanding the relationships between doctors and their patients and how this affects patient health outcomes [1-7]. However, little if any attention has been given to the ways in which doctor-patient relationships may vary across different practices and different locales. While the basic script may remain the same [7-12], the nature and type of communications and interactions between doctor and patient may differ across practice locations, such as urban and rural settings. If doctors and patients interact differently in urban and rural settings, then patient care and ultimately patient health outcomes could differ as a result.

Research has shown that communication, as an interactional process involving both doctors and patients, is key to understanding the quality of care patients receive [1,2,5-7,13]. Communicating and exchanging information are key to diagnosis and treatment [3,14-17]. It is also essential in building a trusting relationship between doctor and patient that encourages better information-giving from patients and better information-getting from doctors, both of which are particularly important when doctors have limited amounts of time with their patients [15,16,18,19]. Furthermore, communication and trust may influence patient satisfaction with care, patient compliance, how patients cope with their health concerns, and potentially patient health outcomes [3,6,14,20].

This paper explores whether doctor-patient communications differ across rural and urban family clinics. Based on observation and interview data with two family physicians from each setting, we illustrate subtle but important differences in doctor-patient interactions in rural and urban clinics.

## Methods

This is an exploratory pilot study using interview and observation data from four physicians working in either rural or urban family clinics. These four participants were part of a larger study on physician wellness that included direct observations and interviews with 42 physicians working in a single health region in Western Canada. The health region includes a major urban centre of approximately one million residents and its surrounding areas. The four physicians were purposefully selected to represent typical “information-rich cases” [21], which allows us to study the communication patterns of rural and urban doctors and their patients in greater depth and detail. The two rural physicians were selected for this analysis because they were the only rural family doctors in the larger sample, while the two urban physicians were selected because they were the most information-rich relevant cases. Prior to this research project, none of the physicians had any relationship with the second author, who is a female professor of sociology, and who carried out the interviews and observations.

First, the physicians were interviewed at their place of work. The interviews consisted of mostly open-ended questions where physicians were asked to describe their work and non-work experiences, sources of satisfaction and stress, coping strategies, and provide demographic and practice information. The interviews ranged in length from approximately 20 to 40 minutes, averaging about half an hour in duration. The interviews were face-to-face and digitally recorded. At the end of the interview, a time was scheduled for a half-day of direct observations, or job shadows, with each physician. While the purpose of the observations was to observe physicians during their typical workday, this paper focuses on the communication styles utilized by physicians during these observations. Their daily activities and patient interactions are often so familiar and taken for granted that it makes it difficult for them to identify, describe or explain them in an interview or questionnaire. The researcher, as an outsider to the medical profession, entered the settings with no preconceived notions of what to observe, but rather with the intent to explore and describe the experiences of practicing medicine. The observer was a non-participant during the doctor-patient encounters. Observation notes were recorded into an electronic tablet that were then edited and transcribed immediately following the job shadow. Observations lasted from approximately 3½ to five hours in duration. The field notes and other information were not presented to the participants for comments.

Written informed consent was given before interviews and observations were completed and all study participants knew their participation was voluntary and they could withdraw at any time. In order to protect the anonymity of the participants, pseudonyms have been used. The two urban doctors have been re-named as Dr. Jim Barrett and Dr. Mary Cummings, and the two rural doctors have been re-named as Dr. Alan Jones and Dr. Colleen Walker. Ethics approval for this study was obtained from the Conjoint Health Research Ethics Board of the University of Calgary’s Faculty of Medicine.

### Participants and clinics

The two urban doctors, Dr. Jim Barrett and Dr. Mary Cummings (pseudonyms), have practiced medicine for about twenty years. They worked in family clinics located in “strip” malls in middle/upper class neighbourhoods that primarily consisted of single family homes. In addition to the clinics, these neighbourhood malls also contained other small businesses, such as a convenience store and gas station, dry cleaner, restaurant, and hair salon. Patients attending these clinics had appointments scheduled in advance and usually had seen the physician previously. Many of the patients lived in the surrounding community where the clinic was located. The observed appointments involved such activities as refilling prescriptions, baby checks, reviewing test results (e.g., ultrasound, bloodwork), follow-ups for pre-existing conditions (e.g., rash, wart, blood pressure, blot clots, fibromyalgia), referrals, and physical exams. The average length of patient visits was approximately 11 minutes, although they varied significantly ranging from 4 to 20 minutes.

Both rural physicians, Dr. Alan Jones and Dr. Colleen Walker (pseudonyms), have practiced medicine for eighteen and twenty-two years respectively, and were both working in group family practices. The rural family clinics were located in two small towns – one with a population of less than 2,000 and the other less than 10,000. The primary industries in these communities are agriculture and natural resources. Patients booked their appointments in advance and often included several family members (e.g., spouses, parents or children). The rural physicians seemed to see their patients quite regularly and if a non-urgent health concern was raised on the way out, the doctor often said they could talk about it next time. The health concerns presented to these rural physicians included referrals, prescription refills, reviewing test results (e.g., MRIs, blood work, biopsies), taking biopsies and conducting physical exams. The visits averaged approximately 12 to 13 minutes in duration, although some were as short as 3 minutes and others as long as 30 minutes.

### Data analysis

Utilizing a grounded theory approach [22], and a line by-line-coding strategy exercised by one coder, the first author, a doctoral candidate with graduate training in grounded theory, several themes emerged from the observation data until saturation was reached and nothing new emerged. The themes indicated different types of interactions displayed between doctors and patients. These themes were also detected in the interview data. After the themes were identified, bearing in mind that “all is data”, the authors searched the literature for more information on doctor-patient communication styles, which further informed the conceptual labels developed and used in this paper (Glaser, 2001 [23]: 145).

## Results

Analysis of the observation and interview data yielded two different patterns of interactions between doctors and patients that were consistent with ones previously identified in the literature. Specifically, these included instrumental communication and socioemotional communication [3,5]. *Instrumental communication* refers to “cure-oriented interactions” [2] where the doctor and patient discuss the health concerns or reason for the appointment and share information that is directly related to the patient’s physical health [3]. It involves information giving and question asking by both patient and physician with the primary goal of treating the patient’s illness and health concerns [5,6]. The content of instrumental communication often includes the physician asking about symptoms, recording information in the patient’s chart, explaining tests or illnesses, and prescribing and explaining medications. *Socioemotional communication* refers to “care-oriented interactions” [2] that have the primary goals of making the patient feel comfortable, relieving patient anxiety and building a trusting relationship [3,5]. It may involve positive talk where the physician expresses friendliness, empathy, sympathy, concern, reassurance and partnership building. The specific elements of socioemotional communication may include greeting the patient in a friendly way, addressing the patient by name, engaging in small talk, being friendly, and listening attentively. Three specific types of interactions were central to the socioemotional communications observed in this study: the use of informal pleasantries, the use of humour and active relationship building. *Informal pleasantries* often occurred at the beginning of the interaction and refers to casual communication that the doctor and patient engage in without any advanced knowledge of each other, such as discussing the weather or a recent sporting event. *Humour* during the interaction was also observed and involves sarcasm, teasing and joking from either the doctor or the patient. Laughing and making jokes has been identified as a necessary ingredient of good inter-personal relationships between doctors and patients [3]. *Relationship-building* refers to communication involving any personal talk about the patient’s and/or physician’s life outside the medical office and their roles in it. Table 1 provides examples of instrumental and socioemotional communication observed in the four cases examined in this study.

**Table 1:** Descriptive information on observations and examples of instrumental and socioemotional communication

**Setting (Physician)**	**# Patients and visit duration**	**Examples of instrumental communication**	**Examples of socioemotional communication**
**Urban (Mary)**	19 patient visits observed	● Patient asks about test results	● Greet each other by their first name
270 minutes of observation	Average of 11 minutes per visit	● Patient asks for refills	● Patient asks about physician’s daughter and what grade she’s in now
	(range 5 to 20 minutes)	● Patient asks when test results will be back	● Patient teases physician about being tanned from recent vacation
		● Physician asks about symptoms	● Physician asks about how patient’s sibling is coping
		● Physician explains tests being scheduled for patient	● Patient and physician talk about people they both know who are retiring
		● Physician explains prescriptions and side effects	● Physician teases patient about smoking
		● Physician reviews test results	
**Urban (John)**	18 patient visits observed	● Patient describes symptom	● Greet each other by first name
220 minutes of observations	Average of 11 minutes per visit	● Patient asks about health coverage of tests, medications	● Patient jokes that he is there because his wife told him to come in
	(range 4 to 19 minutes)	● Patient asks for refills	● Physician teases patient about being an “empty nester”
		● Patient asks about their self diagnosis (e.g., thyroid problem, appendix, cancer)	● Physician asks about patients’ parent’s recent surgery, about son in overseas, say “hi” to dad
		● Physician explains medications and side effects	● Physician ask how patient’s recent trip was traveling with babies
		● Physician asks patient if s/he has any questions about treatment or prescriptions	
		● Physician explains tests being scheduled for patient	
		● Physician reviews test results	
**Rural (Alan)**	23 patient visits observed	● Patient describes symptoms and health concerns	● Greet each other by first name
275 minutes of observations	Average of 12 minutes per visit	● Patient asks about health coverage of treatment	● They joke about aging, failing knees, bunnies do well eating salads, giving up coffee, golf not being exercise
	(range 3 to 19 minutes)	● Patient asks about prescriptions and side effects	● Talk about summer vacations, exercise routine, daughter getting married, grandson in hockey tournament, restoring cars, hockey game last night, family conflicts and estrangements
		● Physician reviews test results	● Physician teases patient about drinking too much at local weekend party
		● Physician explains tests being scheduled for patient	● Doctor refers to some patients as “My Dear”
		● Physician explains health concerns	● Physician asks patient how he’s doing and pats patient’s knee
		● Physician asks about symptoms	● Physician talks to teens about sex, tattoos, drinking and drugs
**Rural (Colleen)**	13 patient visits observed	● Patient asks about tests and referrals	● Greet each other by first name
297 minutes of observations	Average of 13 minutes per visit	● Patient describes tests and referrals	● They joke about patient falling off examining table and a good looking doctor to come help her up, about weight loss, quitting smoking,
	(range 4 to 30 minutes)	● Physician asks about symptoms	● Talk about deer eating flowers, each others’ daughters, retirement celebration for a physician in the clinic, parent who is recently widowed, vacation, sister’s health, new haircut
		● Physician explains test results	
		● Physician explains procedures	
		● Physician explains health concerns	

### Urban doctors

The urban doctors, Dr. Jim Barrett and Dr. Mary Cummings, displayed socioemotional communication with some of their patients, but at least half of the appointments were purely instrumental in nature. An appointment was considered “strictly instrumental” when both the physician and patient only discussed the medical reason for the visit without engaging in other more personal, non-medical communications. That is, these appointments involved discussion of the patients’ ailments and potential treatment without any socioemotional communications such as informal pleasantries, humour, or relationship-building. For example, 12 of the 18 patient visits observed for Jim and 13 of the 19 visits observed for Mary were strictly instrumental in that they did not engage in any socioemotional communication.

In contrast, in other patient visits in the urban clinics, informal pleasantries (e.g., discussion about the weather) were exchanged during the course of the appointment and the doctors referred to their patients by their first name. The urban doctors asked some medical history questions of the patients and appeared to have general knowledge of many of their patients’ day-to-day lives and could speak to that on a more personal level rather than just to their immediate health concerns. Socioemotional communication and relationship-building, however, appeared to depend on the specific patient involved. For example, Jim left a solo downtown practice for a group suburban one, and many of his patients followed him from his downtown practice. One of the patients he saw during the course of the observation had recently bumped into Jim at a nearby restaurant. Many patient-interactions, however, were purely instrumental where the majority of the appointment was devoted to discussing the reason the appointment was scheduled.

When asked about her primary duties, Mary did not include descriptions of taking medical histories or reviewing the charts as her primary work tasks. Rather, she drew attention to the time she spends specifically seeing patients and getting to know them. Jim responded similarly to the same question: “Well most of my time is patient care.” When probed for more information, he explained further:

“I mean on a typical schedule day we tend to slot patients in for 10 minute appointments but that’s for individual issues, individual visits for doing oh medicals and things, I usually allot a half an hour for those to give us a bit more time.”

When asked what she enjoys most about her job, Mary answered: “I enjoy getting to know my patients.” Mary finds her patients to be the most satisfying part of her job, yet her enjoyment does not end with merely spending time with patients, it is about “getting to know” them. Jim, the other urban doctor, stated: “You know, I like spending time with patients, I like talking with patients but on an economic basis you just can’t afford to do that.”

This comment suggests that while Jim enjoys spending time with patients, talking to patients and getting to know them on a personal level, he feels limited in how much time he can afford each patient because economically it is not viable. His comments are consistent with the patterns that emerged during observations. While the urban doctors spent time cultivating supportive ties with their patients, these were somewhat restricted interactions and they did not typify every appointment. Later in the interview, Jim explained why he enjoyed spending time with his patients:

“Um, getting to know them and after many years, not just getting to know them in a professional sense, but I mean I lived very close to where my office was for the bulk of my career and many of my patients’ kids actually went to school with my kids… so that’s part of the reason I sometimes get behind is now we’re at the point where talking about what’s [doctor’s kid’s name] up to today and where is he going to school and so and so is getting married and various kinds of things… that to me is very satisfying.”

Jim expressed his desire to establish long-term relationships with his patients. He wants to engage with his patients in a community setting rather than merely doing “medicine, medicine, medicine, medicine” and simply moving from one patient to the next during his work day.

### Rural doctors

The observation data showed that both rural doctors interacted with their patients on a very personal level, and talked with their patients about their personal, medical and social lives as well as those of their family and friends. Very few of the patient visits with the rural doctors were strictly instrumental. In fact, only one of the 23 patient visits observed for Alan and only two of the 13 visits observed for Colleen were considered purely instrumental without any socioemotional communication.

During most visits, both doctors and their patients usually chatted about non-health related topics in terms of personal matters and activities, and community events that reflect how these socioemotional relationships were cultivated between patient and physician in addition to patients’ health-related concerns. These appointments drew on both socioemotional and instrumental communications, thereby reflecting more blended communication styles. For example, Alan talked to his patients about such topics as their summer vacations, children’s marriages, grandsons’ hockey games, hobbies, renovations, and gardening. He also shared some very personal aspects of his life with his patients during their appointments. Colleen often checked on her patients’ emotional and mental wellness to see how they are coping with any personal difficulties they were experiencing, such as ailing parents or difficult children. In many patient encounters, Colleen and her patients’ faces visibly lit up when they saw each other, like old friends seeing one another, and they would almost pick up on an ongoing conversation as if it had been interrupted only a few minutes ago.

Alan, coaches a local youth sports team, which provided an easy topic to discuss life apart from the office and offered a way to introduce informal pleasantries. Additionally, both rural doctors frequently called patients by their first names, nicknames, or even pet names, such as “My Dear”. In addition, some of the patients referred to the doctors by their first names.

When asked about their general duties and work tasks, Alan answered quite plainly: “Seeing patients.” The urban doctors elaborated on what seeing patients entailed, but Alan saw no need to do so, nor did he mention the amount of time he spends doing paperwork or other office tasks. Like the urban doctors, he did not mention needing to take past medical histories and the like, probably because he was more familiar with his patients and their day-to-day lives. In contrast to Alan, Colleen, described the administrative responsibilities that she is also responsible for in addition to her family practice, and did not discuss patient care in much detail.

When asked about the most satisfying thing about his work, Alan answered: “Well patient contact. There’s ah yah, that’s why I’m in general practice or family medicine.” Colleen responded similarly: “The patient interactions.” Like the urban doctors, the rural doctors found spending time with patients to be the most satisfying part of practicing medicine. However, when asked what she found most rewarding about her work, Colleen explained:

“Um, [p] the most rewarding? Um the patient interaction… But it, and so it’s the relationship, um fostering a patient to be able to do a lot of self care, that type of thing, that’s good.”

Here, Colleen draws attention to how rewarding it is to get to know her patients better and help them engage in self care and self management practices.

Everyone in these communities knows everyone else and patients keep the physician up-to-date on what is going on in their lives and in the community. It appears that there is an immense amount of trust and caring in these relationships that exhibited considerable socioemotional communication and relationship building, where patients not only disclosed physical health matters but also raised more personal concerns about their spouse, parents or children.

## Discussion

This paper explored whether doctor-patient interactions differ across urban and rural clinics. The observation and interview data suggest that differences exist in doctor-patient interactions in different locales. Of the four doctors that we studied, those who live and practice in small rural towns appeared to know their patients and their families on a more personal level and outside of their office, and engaged in more socioemotional communications compared to those who live and practice in suburban clinics a large urban centre. Knowing patients outside the clinic seems to change the nature of the doctor-patient interaction, and, in turn, the doctor-patient relationship itself.

Instrumental communication was the basis of more of the doctor-patient interactions observed in the urban settings, whereas rural doctors and patients exercised more blended communications that drew on both socioemotional and instrumental styles. Socioemotional communication and relationship-building typified most of the interactions observed in rural settings although these interactions also contained instrumental components, and were also seen in many of the urban doctor-patient interactions. Informal pleasantries were more often used in urban clinics, but rural doctors used them as well. Humour was used in both settings, but was more prevalent in rural ones. While communication styles differed across settings, it is interesting to note that the average amount of time allotted for purely instrumental visits was the same as that devoted to blended visits involving both instrumental and socioemotional communication. That is, even though the urban doctors were more likely to engage in strictly instrumental interactions with their patients, their appointments that also included socioemotional communication averaged about the same length of time. This is important to consider particularly in light of Becker et al’s (2010 [15]) and Wiegl et al’s (2009 [19]) findings that patients feel they spend little time with doctors. Building rapport with patients is necessary to make the most of these minutes, and quickly developing a trusting relationship can help physicians make the most of that time.

Rural doctor-patient interactions observed in this study involve discussion of many personal, family and community topics unrelated to the health concern. Informal pleasantries are exchanged, and much joking and bantering occurs throughout the interaction. Both patients and physicians refer to each other with terms of familiarity and endearment. These relationships are clearly long-term, trusting and personal. Each knows intimate information about the other and their families as “everybody knows everybody” in these small, rural communities. These doctor-patient interactions are characterized by considerable socioemotional communication and relationship-building.

The urban interactions involved informal pleasantries as patients and physicians checked in with one another at the beginning of the visit. This small talk was usually not as personal nor intimate as that observed in the rural clinics. The interactions were also supportive and relationship building, however, in that the physician may have asked about other family members during the visit in regards to non-health related topics. Again, these urban conversations, while personal and emotionally supportive, were not to the same extent as those observed in the rural clinics. The urban interactions tended to be more instrumental and task oriented than those in the rural clinics where most of the conversation in the urban clinics was focused on the patients’ health concerns.

As indicated above, differences in doctor-patient interactions may affect patients’ health and future health outcomes. A growing body of literature suggests that socioemotional communication with ones’ physician positively influences individual health outcomes [3,5-7,14,17]. A socioemotional relationship with one’s doctor may enhance patient health through establishing a trusting relationship where anything can be shared. However, it could also be detrimental through an assumed understanding and knowledge of a patient’s condition. Doctors who feel they know their patients on a more personal basis, and who assume they are more familiar with the patient’s medical background, may not take as thorough a history at each visit compared to doctors who rely more on instrumental communication during patient visits. More research should examine the relationships between different types of communication involving physicians and patients and the health of patients and quality of healthcare offered at different sites.

In addition, future research might consider rural doctor-patient relationships and healthcare outcomes, particularly as rural health could be adversely affected by shortages of physicians and medical practices in these areas. Potentially, rural residents with family doctors could have very different health outcomes compared to rural residents without family doctors, urbanites with family doctors, and urbanites without family doctors. Research that compares these groups could provide a better understanding of the impact of doctor-patient relationship on patient health and healthcare outcomes.

There are several limitations to this research. Because of the exploratory nature of this pilot study, the sample size is small, thus raising concerns about generalizability. In other words, the themes found based on the data for these four physicians may not be generalizable to the greater population of urban and rural family doctors. The transferability or usefulness of these results to practice may be limited until a larger sample size of both urban and rural family doctors are observed. Additionally, while saturation was reached with the limited number of observations in this study, a larger sample of physicians may generate more themes than those discovered in this project. Further, as in all observational research, the data were restricted to that which the particular field researcher drew attention to and noted in her observations. In this case, the researcher was an “outsider” in the research setting, that is, she did not have a background in practicing medicine. While “insider” perspectives such as medical professionals observing medical practice settings, can be beneficial as insiders may better understand the nomenclature and culture of what they are observing, “outsider” perspectives of those relatively unfamiliar with the phenomena being studied may be better able to observe the taken for granted [24]. It should also be recognized that small towns located nearer to major citites, such as those included in this study, are very different from small towns in more remote locations [25]. Our dichotomy of rural–urban does not capture the varying degree of rurality of communities, particularly those in remote locations. Last, consultation time may not fully reflect the entire domain of doctor-patient interactions. Physicians and patients may also interact outside of patient visits that occur beyond the clinics, for example through phone calls, mail, or face-to-face encounters in the community. As well, most of the appointments observed in this study were extensions of already established relationships between the doctors and their patients, and represent only a single interaction within an ongoing cycle of care [4]. Additionally, because the authors did not access patient histories or ask the physicians how often they saw each patient, it is impossible to account for the development of physician-patient interactions outside the observed encounters during patients’ appointments in the clinics. Familiarity outside of patient visits could also enhance the use of socioemotional communication within these interactions.

## Conclusions

The results of this study show that the doctor-patient interactions observed in the urban settings were primarily instrumental, or “cure-oriented interactions”, that were oriented towards the health concerns or reason for the appointment and information that was shared was directly related to the patient’s physical health [3]. In contrast, the results suggest that doctor-patient interactions in rural settings involved significantly more socio-emotional communication that was characterized by more “care-oriented interactions” [2]. These socioemotional communications may ease patient anxiety and increase patient-doctor trust, but they did not appear to add extra time to the patient visit. In these rural interactions, medicine happened in between conversation that may alleviate anxiety and builds a trusting relationship. An interesting and somewhat unexpected finding of this study is that despite the different ways in which doctors and patients communicated with each other, for urban doctors, purely instrumental and blended appointments averaged approximately the same amount of time. Thus, it does not appear that building trusting, personal relationships with patients while garnering vital instrumental information resulted in lengthening the time of patient appointments.

Research suggests that socioemotional communication ultimately leads to better patient outcomes [3,5]. This implies that health differences between rural and urban settings could be linked to differences in doctor-patient communication styles. And while some might argue that socioemotional communication takes up too much time, the results of this study suggest otherwise. While uncertainties remain about the role of physician communication style and patient outcomes [25,26], by better understanding the different types of doctor-patient communications and how they vary across urban and rural settings, it may provide an important piece to the complex puzzle of explaining urban–rural variations in health care and health outcomes.

## Abbreviations

CA: Conversation analysis; AHS: Alberta health services; AHFMR: Alberta Heritage Foundation for Medical Research.

## Competing interests

The authors declare that they have no competing interests.

## Authors’ contributions

JW was involved in the design and implementation of the study. JW completed the observations and interviews of the physicians that contributed to this study while KDD, supported by JW, completed the first wave of analysis for this paper and completed the first draft of the manuscript. Both authors contributed to the manuscript, and have read and approved the final manuscript.

## Pre-publication history

The pre-publication history for this paper can be accessed here:

http://www.biomedcentral.com/1472-6963/13/261/prepub
